# GRAF1 Regulates Brown and Beige Adipose Differentiation and Function

**DOI:** 10.21203/rs.3.rs-3740465/v1

**Published:** 2023-12-19

**Authors:** Xue Bai, Qiang Zhu, Matthew Combs, Martin Wabitsch, Christopher P. Mack, Joan M. Taylor

**Affiliations:** University of North Carolina at Chapel Hill; University of North Carolina at Chapel Hill; University of North Carolina at Chapel Hill; Ulm University Medical Center; University of North Carolina at Chapel Hill; University of North Carolina at Chapel Hill

**Keywords:** GRAF1, adipose tissue, Browning, thermogenesis, energy metabolism

## Abstract

Adipose tissue, which is crucial for the regulation of energy within the body, contains both white and brown adipocytes. White adipose tissue (WAT) primarily stores energy, while brown adipose tissue (BAT) plays a critical role in energy dissipation as heat, offering potential for therapies aimed at enhancing metabolic health. Regulation of the RhoA/ROCK pathway is crucial for appropriate specification, differentiation and maturation of both white and brown adipocytes. However, our knowledge of how this pathway is controlled within specific adipose depots remains unclear, and to date a RhoA regulator that selectively controls adipocyte browning has not been identified. Our study shows that expression of GRAF1, a RhoGAP highly expressed in metabolically active tissues, closely correlates with brown adipocyte differentiation in culture and in vivo. Mice with either global or adipocyte-specific GRAF1 deficiency exhibit impaired BAT maturation, reduced capacity for WAT browning, and compromised cold-induced thermogenesis. Moreover, defects in differentiation of mouse or human GRAF1-deficient brown preadipocytes can be rescued by treatment with a Rho kinase inhibitor. Collectively, these studies indicate that GRAF1 can selectively induce brown and beige adipocyte differentiation and suggest that manipulating GRAF1 activity may hold promise for the future treatment of diseases related to metabolic dysfunction.

## Introduction

Adipose tissue is comprised of two major cell types- white and brown adipocytes-that have opposing energetic functions. White adipocytes primarily function to store excess lipid and an over-abundance of white adipose tissue (WAT) leads to unfavorable outcomes due to disruption in many metabolic and hormonal activities. In contrast, brown adipocytes consume glucose and lipid and brown adipose tissue (BAT) dissipates energy through uncoupled oxidative phosphorylation-induced heat generation, a process necessary for thermoregulation in small animals and infants. Interestingly, in response to various environmental cues, including following cold exposure, a subset of adipocytes within WAT undergo browning and these so-called “beige” fat cells exhibit many of the beneficial metabolic features of classical brown adipocytes including dissipation of energy through thermogenesis and the secretion of endocrine factors that promote whole body metabolism. As obesity results from a disparity between energy intake and expenditure, the identification of new molecules and signals that can drive fat browning may lead to attractive targets for the treatment of obesity and related cardiometabolic disorders.

WAT and BAT are located in discrete depots that are distributed throughout the body and are derived from a diverse group of progenitor cells. While we do not yet know the precise origins of cells within each of these tissues, current lineage tracing studies indicate that Myf5-expressing progenitor cells that originate in the dermomyotome give rise to greater than 90% of inter- and sub-scapular BAT (iBAT and sBAT) which are the largest BAT depots in mice^1, 2^. Other adipose depots likely arise from a more heterogeneous population of progenitors. For example, the contribution of Myf5 progenitors to WAT varies tremendously (from 5 to 60%) based on anatomic locale and sex^1^. The mural cell compartment of the vasculature also appears to be a major contributing source of adipogenic precursors in various WAT compartments.

Despite their heterogeneous origins, white and brown adipogenic precursors share some common transcriptional programs. For example, peroxisome proliferator–activated receptor gamma (PPARγ) is a master regulator of adipocyte differentiation and this transcription factor drives expression of a number of genes common to all adipocytes including adiponectin and fatty acid binding protein 4 (FABP4) ^3, 4^. Moreover, classical brown adipocytes that originate from the dermomyotome and beige adipocytes that are intermingled in WAT depots share PGC1α- and PRDM16- dependent transcriptional programs that drive mitochondrial biogenesis and induce a common subset of BAT-selective genes. These include, among others, Cidea which promotes lipolysis and lipogenesis and uncoupling protein 1 (UCP-1) which is responsible for facilitating thermogenesis through uncoupled respiration ^5^.

Importantly, both white and brown adipocytes undergo dynamic and reversible phenotypic conversions in response to environmental cues. For example, thermoneutrality or exposure to high fat diet can induce BAT to undergo reversible WAT-like remodeling. Conversely, long-term cold exposure, agonist-induced activation of the β3 adrenergic pathway, or physical activity can promote WAT to undergo browning. The beneficial physical activity-induced phenotypic changes are mediated, at least in part, by skeletal-muscle derived factors including fibroblast growth factors(FGFs) and bone morphogenic proteins (BMPs) ^6, 7^.

BMP 4, 6, and 7 are particularly strong inducers of brown and beige adipogenesis and, while we do not fully understand the underlying signaling mechanisms, downregulation of RhoA-mediated signaling has been shown to be important ^8–12^. For example, down-regulation of RhoA in white adipocytes promotes beige cell development and heterozygous germline depletion of the RhoA-dependent kinase, ROCK2, or treatment with Rho kinase inhibitors promoted WAT browning and led to protection from diet-induced obesity and insulin resistance in mice ^13^. Interestingly, the dynamic regulation of RhoA is also important to control the fate of mesenchymal stem cells and multi-potent progenitors wherein low RhoA activity favors pre-adipocyte specification while high RhoA activity promotes specification towards osteoblast, smooth- or skeletal muscle cell fates ^14^. Mechanistically, RhoA/ROCK signaling promotes these cell fate conversions by modulating both actin cytoskeleton-dependent shape changes and altering gene transcription (RhoA activity inhibits the pro-adipogenic transcription factor, PPARγ- and activates myogenic SRF/MRTFA- mediated gene transcription) ^15, 16^.

RhoA, like all small GTPases, cycles between an inactive GDP-bound form and an active GTP-bound form and its activity is enhanced by guanine nucleotide exchange factors (GEFs) and inhibited by GTPase-activating proteins (GAPs). To date, two RhoA-specific GAPs have been identified as critical regulators of adipogenesis, p190B and DLC1. P190B limits RhoA activity in myogenic precursors and is necessary for these cells to adopt a pre-adipocyte fate^17–19^. On the other hand, DLC1 limits RhoA activity in pre-adipocytes and functions to promote white and brown adipogenesis ^20^. However, a GAP that selectively controls adipocyte browning has not yet been identified. Herein, we show that the multidomain containing RhoA-GAP termed GRAF1 (guanosine triphosphatase (GTPase) regulator associated with FAK-1, also named Arhgap26) ^21, 22^that was previously reported to be strongly expressed in highly metabolic tissues (including heart, brain, and skeletal muscle) is also strongly expressed in BAT and functions to selectively promote the activation of BAT and the browning of WAT. Our findings highlight the possibility that GRAF1 could be a new therapeutic target to combat obesity and associated morbidity.

## Results

### GRAF1 expression correlates with BAT maturation in mouse and human cells.

We previously reported that GRAF1 was transiently upregulated during skeletal muscle development (E17-P4) and again following adult muscle injury and that, in these contexts, GRAF1 promoted myoblast differentiation by limiting RhoA activity^23^. Interestingly, while dissecting various muscles from adult GRAF1 hypomorphic (GRAF1^gt/gt^) mice, we noticed that their iBAT depots were relatively pale compared to WT controls. Since myocytes and BAT share common Myf5-expressing precursors^1, 18, 24, 25^ and both cell types require RhoA inactivation for differentiation and maturation^26^,we further investigated a possible role for GRAF1 in BAT development.

**Figure 1:**
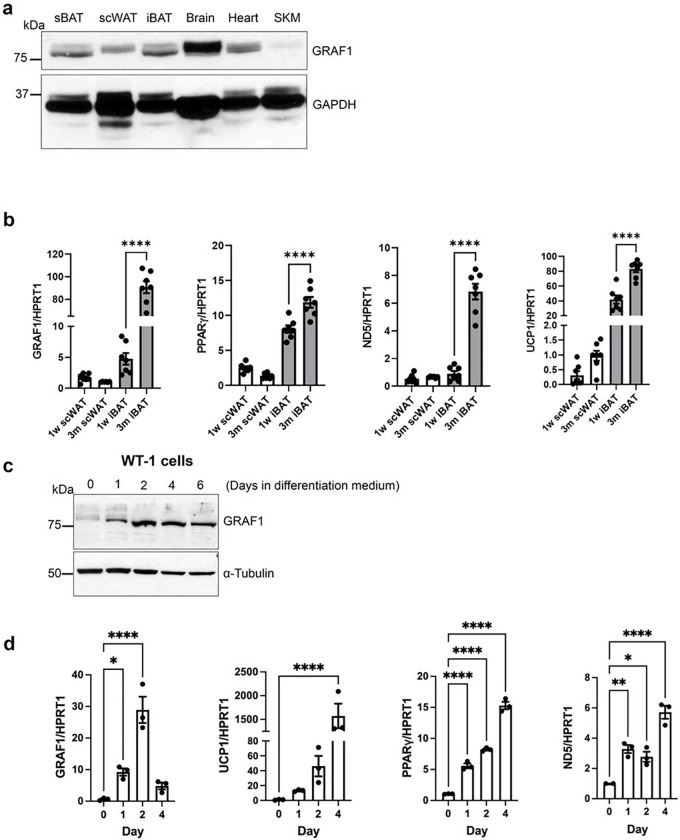
GRAF1 expression closely correlates with BAT maturation. **a.** GRAF1 protein levels in 3 week postnatal mouse tissue detected by Western blotting. scWAT, sub-cutaneous white adipose tissue; iBAT and sBAT, inter- and sub-scapular brown adipose tissue, SKM, skeletal muscle. **b**. qRT-PCR of GRAF1 and adipocyte marker genes in indicated adipose depot isolated from 1 week old and 3 month old mice, n=6–7. **c,d.** WT-1 pre-brown adipocytes were exposed to brown fat-inducing insulin differentiation medium for the indicated times followed by Western blotting (**c**) or qRT-PCR (**d**) analysis of GRAF1 or indicated adipocyte markers (n=3). Data(**b,d**) are represented as mean ± SEM, *P < 0.05; **P < 0.01; ****P < 0.0001 by two-tailed student’s t-test.

In mice, iBAT and sBAT depots develop during late embryogenesis, and, upon expression profiling in juvenile mice (3 weeks postnatal), we found that GRAF1 was highly expressed in these major BAT depots. GRAF1 was also expressed (albeit at a lower level) in subcutaneous WAT (scWAT) (Fig. 1a). Interestingly, GRAF1 mRNA levels in iBAT increased nearly 20-fold from 1 week to 3 months of age (Fig. 1b) and this increase paralleled the expression of BAT maturation genes including PPARγ, the thermogenic protein UCP1, and the mitochondrial marker ND5. In contrast, GRAF1 levels did not significantly change during WAT maturation.

We next used cultured cell models to confirm and extend these findings. First, using WT-1 cells (a validated in vitro model of brown adipogenesis)^27^, we found that GRAF1 expression was dynamically and transiently increased at the onset of differentiation (Fig. 1c,d) and that its induction occurred prior to, or concomitant with, expression of BAT marker genes UCP1, PPARγ, and ND5 (Fig. 1d). This finding is consistent with prior reports indicating RhoA/ROCK signaling is down-regulated upon the induction of adipocyte differentiation in these cells. A similar dramatic increase in GRAF1 expression was observed in human preadipocytes (Simpson-Golabi-Behmel syndrome, SGBS) following exposure to brown adipocyte differentiation medium (Figure S1a). Note that adipogenic differentiation of these cells was accompanied by lipid droplet accumulation and characteristic brown phenotype observed using light microscopy (Fig S1a, bottom panel). Finally, in mouse 3T3L1 adipocytes, which can be induced to form WAT or BAT, treatment with BAT-induction cocktail led to a more robust increase in GRAF1 expression than did treatment with a WAT-induction cocktail (Figure S1b,c). Collectively, these studies indicate that GRAF1 might play an important and conserved role in promoting BAT development.

### GRAF1 is necessary for BAT maturation and function

**Figure 2:**
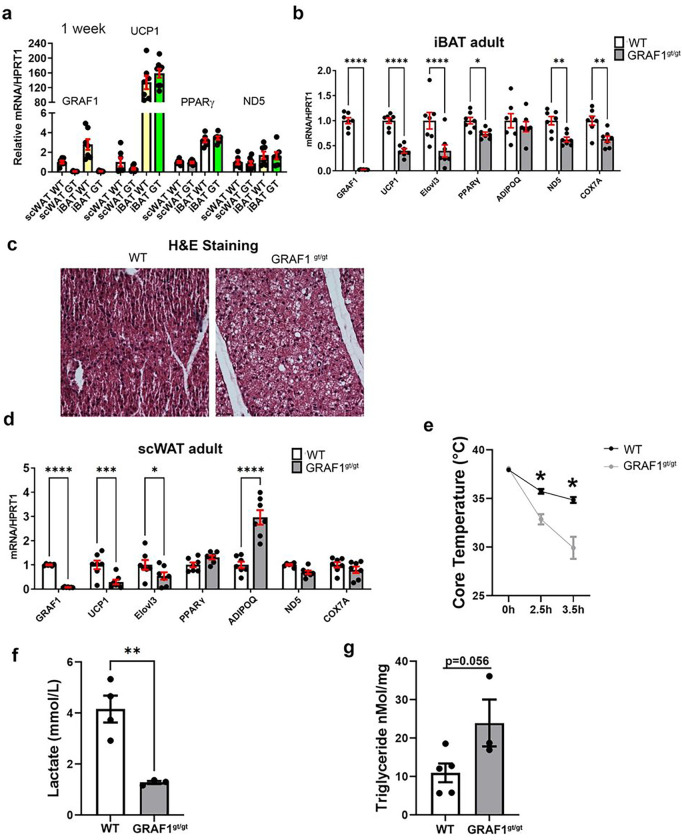
GRAF1 does not alter adipocyte specification but is required for brown fat formation and function. **a**. iBAT and scWAT isolated from 1 week old WT and GRAF1^gt/gt^ mice exhibited similar levels in adipose marker gene expression as assessed by qRT-PCR (n=6–9/group), indicating that GRAF1 is not necessary for white or brown adipose tissue specification. **b**, iBAT isolated from 3 month old GRAF1^gt/gt^ mice exhibited significantly lower levels of brown fat marker genes that mediate thermogenesis compared to littermate control mice as assessed by qRT-PCR (n=7). **c**, Representative H&E stain of iBAT isolated from 3 month old GRAF1^gt/gt^ and WT mice. Note increased levels of lipid droplets (un-stained white vesicles) indicative of BAT whitening in GRAF1^gt/gt^ iBAT depot.(n=3/group). **d**, scWAT isolated from 3 month old GRAF1^gt/gt^ mice exhibited significantly higher levels of white fat marker genes and lower levels of beige-fat associated genes compared to littermate control mice as assessed by qRT-PCR. (n=6–7/group). **e-g**, GRAF1^gt/gt^ and WT mice were housed at sub-thermoneutral temperatures (22–25°C) and subjected to overnight fasting followed by an acute bout of cold stress (6°C) and their body temperatures were measured over time using a rectal thermometer(**e**) and serum lactate(**f**)and soleus triglyceride(**g**) level were measured. (n=4–5 WT, n=3 GRAF1^gt/gt^ ). Data(**b,d,e,f,g**) are represented as mean ± SEM, *P < 0.05; **P < 0.01; ***<0.001;****P < 0.0001 by two-tailed student’s t-test.

We next compared BAT and WAT marker gene expression in tissue isolated from WT mice or from global GRAF1-deficient mice (GRAF1^gt/gt^) which harbors the gene trapping vector VICTR48 within the first intron of Graf1. These mice lines are viable and fertile with no obvious abnormalities under baseline conditions 28. As shown in Fig. 2a, we found no differences in expression of UCP1, PPARγ or ND5 in scWAT or iBAT isolated from 1 week old WT and GRAF1^gt/gt^ mice, suggesting that GRAF1 does not play an important role in adipose tissue specialization. However, since adipose tissue maturation happens gradually after birth through young adulthood^29–31^ and GRAF1 expression was robustly increased during this timeframe, we reasoned that GRAF1 might impact adipose differentiation/maturation. Indeed, as shown in Fig. 2b, BAT depots isolated from 3 month-old GRAF1-deficient mice exhibited a dramatic reduction in BAT marker gene expression relative to littermate control WT mice. For example, iBAT from GRAF1^gt/gt^ mice demonstrated a significant reduction in BAT thermogenesis genes (UCP1, Elovl3), mitochondrial genes (ND5, COX7a), and in PPARγ, the master regulator gene of adipocyte differentiation, indicating a differentiation/maturation defect in the iBAT of GRAF1 hypomorphs. Accordingly, H&E staining of iBAT from 2–3 month old GRAF^gt/gt^ mice revealed a “white like” appearance as demonstrated by a substantial increase in lipid deposition and enlarged lipid droplets compared to iBAT from littermate WT control mice(Fig. 2c). scWAT from 2–3 month old GRAF1-deficient mice also exhibited a significant down-regulation of brown marker genes including UCP1 and Elovl3 but upregulation of the general adipose marker adiponectin, suggesting that GRAF1 promotes WAT browning but limits WAT expansion (Fig. 2d). Despite these changes, and consistent with our prior report ^28^, there was no difference in the body weights of GRAF1^gt/gt^ mice and littermate control mice fed ad libitum (data not shown). This finding is consistent with other mouse models that exhibit defects in brown adipose development, yet do not develop obesity, such as those with the loss of the uncoupling protein UCP-1^32^, or the fatty acid metabolism gene Adipose acyl-CoA synthetase-1^33^.

**Figure 3:**
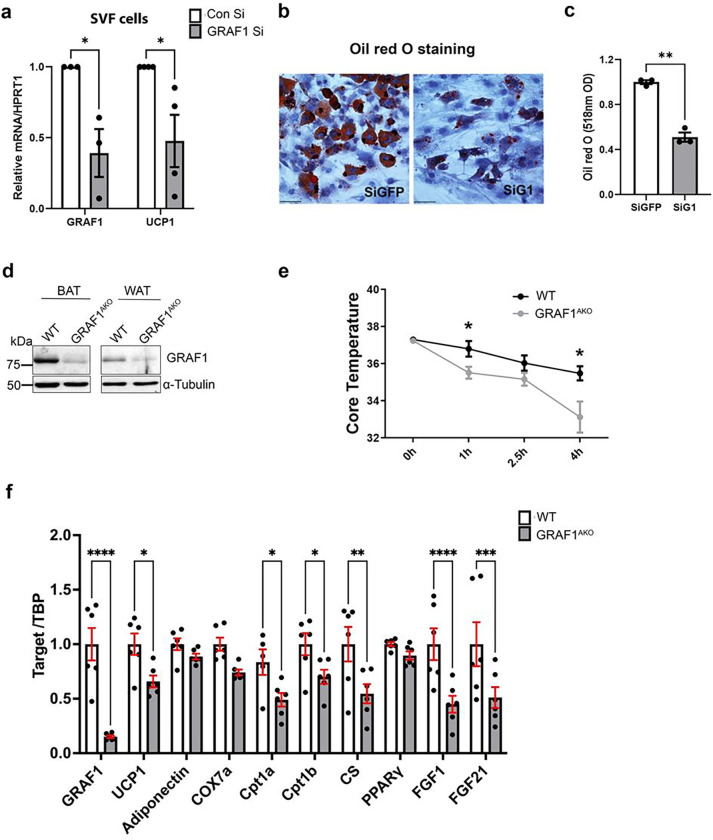
Adipocyte-autonomous role for GRAF1 in BAT formation and function. **a**. differentiation of primary pre-brown adipocytes isolated from the stromal vascular fraction (SVF) of iBAT was reduced by GRAF1 depletion as assessed by UCP1 expression. n=3–4. **b-c**. Oil red O staining of differentiated SVF cells treated with indicated siRNA(**b**) and quantification of oil red O staining(**c**). (siG1 denotes siGRAF1). n=3/treatment. **d**. Depletion of GRAF1 in iBAT and scWAT in WT and GRAF1^AKO^ mice as assessed by Western blotting. **e**. GRAF1^AKO^ mice exhibited cold intolerance when subjected to conditions described in Figure 2e. n=5–6/group. **f**. qRT-PCR data showed significantly decreased markers of brown adipogenesis, mitochondrial components and FGFs in BAT of GRAF1^AKO^ mice. n=6/group. Data(**a,c,e,f**) are represented as mean ± SEM, *P < 0.05; **P < 0.01; ***<0.001;****P < 0.0001 by two-tailed student’s t-test.

To determine the impact of GRAF1-dependent changes in BAT differentiation on BAT function, we next exposed mice housed at sub-thermoneutral temperatures to overnight fasting followed by an acute bout of cold stress (6°C) and measured their body temperatures over time. As shown in Fig. 2e, GRAF1-deficient mice exhibited a remarkable reduction in thermogenic capacity under these conditions. Interestingly, this failure to thermoregulate was associated by a significant reduction in serum lactate levels (Fig. 2f). Since circulating lactate is the main fuel that drives the tricarboxylic acid cycle (TCA cycle) in a fasted state ^34^, the decreased lactate might suggest enhanced TCA cycling due to beta-oxidation defects in GRAF1^gt/gt^ mice. In support of this possibility, there was a trend towards increased muscle triglyceride levels in the cold-exposed GRAF1^gt/gt^ mice relative to similarly treated WT mice (Fig. 2g). Nonetheless, given our knowledge regarding GRAF1’s role in skeletal muscle maturation, we realized that the drop in temperature coupled with the reduced lactate production observed in GRAF1^gt/gt^ mice could be due (at least in part) to a reduced capacity for shivering thermogenesis in these germline-deficient mice. To begin to distinguish between these two possibilities, we first sought to determine if GRAF1 can act in a cell autonomous fashion to promote BAT maturation. To this end, we turned to primary pre-brown adipocyte cultures isolated from the stromal vascular fraction (SVF) of iBAT as these cells have been reported to faithfully recapitulate brown adipocyte maturation when exposed to serum-containing media supplemented with insulin, triiodothyronine (T_3_), dexamethasone, IBMX and rosiglitazone^35^. Importantly, upon induction with differentiation media, SVF cells transfected with GRAF1 siRNA exhibited a significant reduction in browning capacity compared to control siRNA treated cells as assessed by UCP1 expression and oil red O staining (Fig. 3a–c). These findings indicate that GRAF1 levels can directly impact the differentiation of brown adipocytes.

Next, to further explore an adipocyte-autonomous role for GRAF1 in BAT formation and function in vivo, we developed a new mouse model using targeting vectors from Eucomm to conditionally target the GRAF1 allele. In this model, Cre-mediated recombination causes a frame shift and early stop codon (Supplemental Fig. 2a) that results in nonsense mediated mRNA decay. Southern blot analysis confirmed successful targeting in embryonic stem cells (ES) and the germline transmission of resulting chimeras(Tm1a). The Tm1a mice were subsequently crossed with Flp recombinase mice to remove the LacZ reporter and a neomycin resistance cassette used for selection of targeted ES cells to generate GRAF1^fl/fl^(GRAF1^Tm1c^ mice; Supplemental Fig. 2b). Finally, we established an adipose-specific GRAF1 knockout mouse line (GRAF1^AKO^) by crossing GRAF1^Tm1c^ mice with the Adiponectin Cre line (010803, Jackson Lab) ^36^ which led to a significant depletion of GRAF1 in scBAT and WAT (Fig. 3d). Importantly, while not as dramatic as observed in the GRAF1^gt/gt^ mice, the thermogenic capacity of GRAF1^AKO^ mice was significantly blunted when compared to genetic control mice (Fig. 3e). Also, activation of a thermogenic gene expression profile in mature BAT was significantly reduced in GRAF1^AKO^ mice when compared to similarly-treated genetic control mice (Fig. 3f). Consistent with the more modest impact on thermogenesis, while BAT from GRAF1^AKO^ mice exhibited a significant reduction in UCP1 expression, the decrease was not as robust as observed in GRAF1^gt/gt^ mice (possibly due to incomplete recombination in our model). Nonetheless, GRAF1^AKO^ BAT also exhibited significantly reduced expression of the mitochondrial genes Cpt1a, Cpt1b and CS. In addition, GRAF1^AKO^ BAT also exhibited significant reductions in the expression of growth factors known to promote glucose homeostasis including FGF1, which acts in an autocrine fashion to promote glucose uptake in activated BAT and FGF21, an endocrine factor that has been linked to the cardiometabolic benefits of brown fat activation through its ability to promote glucose homeostasis in BAT, skeletal muscle, liver and brain. Collectively, these data confirm that GRAF1 plays an important, adipocyte-autonomous role in BAT formation and function.

### GRAF1 promotes subcutaneous adipose beigeing

**Figure 4:**
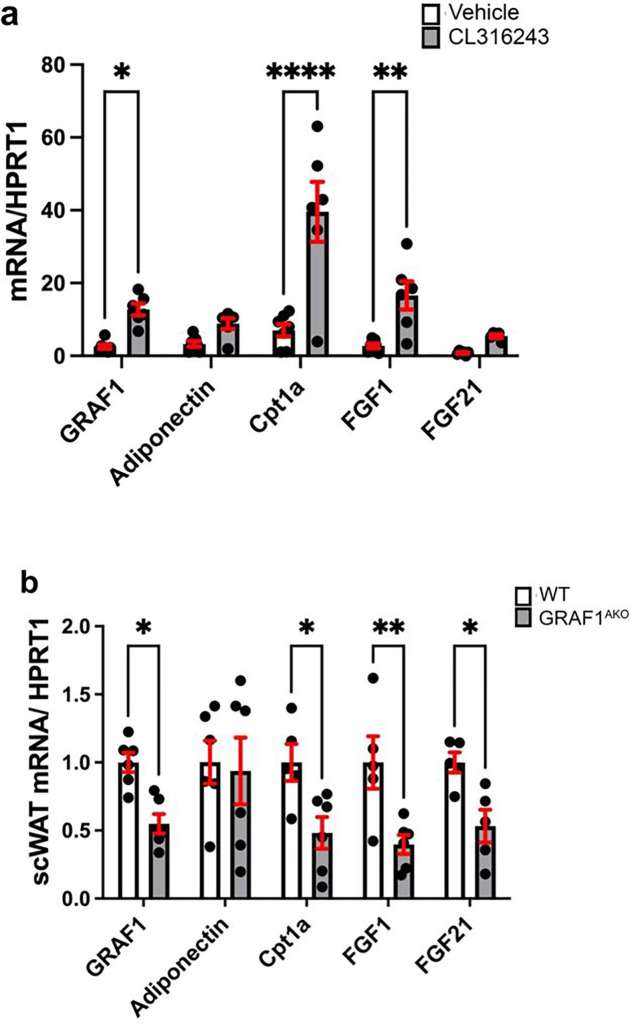
GRAF1 promotes subcutaneous adipose beigeing. **a.** WT mice were treated for 10 days with CL316243, and, qPCR analysis was performed on scWAT to assess mRNA levels of indicated genes; n=5/group. **b**. qPCR analysis of scWAT from WT and GRAF1^AKO^ mice revealed decreased Cpt1a, FGF1 and FGF21 in GRAF1^AKO^ mice. n=5–7/group. Data are represented as mean ± SEM, ns, not significant; *P < 0.05; **P < 0.01 ***<0.001; ****P < 0.0001 by two-tailed student’s t-test.

As noted above, scWAT from adult GRAF1^gt/gt^ mice also exhibited lower BAT marker gene expression relative to similarly housed WT mice, indicating the possibility that GRAF1 might also be necessary for physiological WAT browning. To further explore a role for GRAF1 in beige fat induction, we treated mice with the browning agent CL316243, a β 3-adrenergic receptor agonist. Treatment with CL316234 for 10 days in WT mice promoted expression of several beige adipose markers in scWAT including the mitochondrial enzyme CPT1A (Carnitine Palmitoyltransferase 1) that is essential for fatty acid beta-oxidation ^37^and the secreted factors FGF1 and FGF21 that improve adipose and systemic glucose and lipid metabolism^38^ (Fig. 4a). Interestingly, CL316243 treatment also significantly increased scWAT GRAF1 expression, further suggesting a role for GRAF1 in regulating agonist-induced browning (Fig. 4a). Indeed, the induction of each of these marker genes was significantly reduced in scWAT isolated from CL316243-treated GRAF1^AKO^ mice when compared to scWAT isolated from similarly treated WT mice (Fig. 4b). Thus, in addition to promoting classical brown fat maturation and function, GRAF1 also promotes the development of metabolically favorable beige adipocytes in scWAT.

### GRAF1 promotes brown phenotypes by limiting RhoA/ROCK signaling

**Figure 5:**
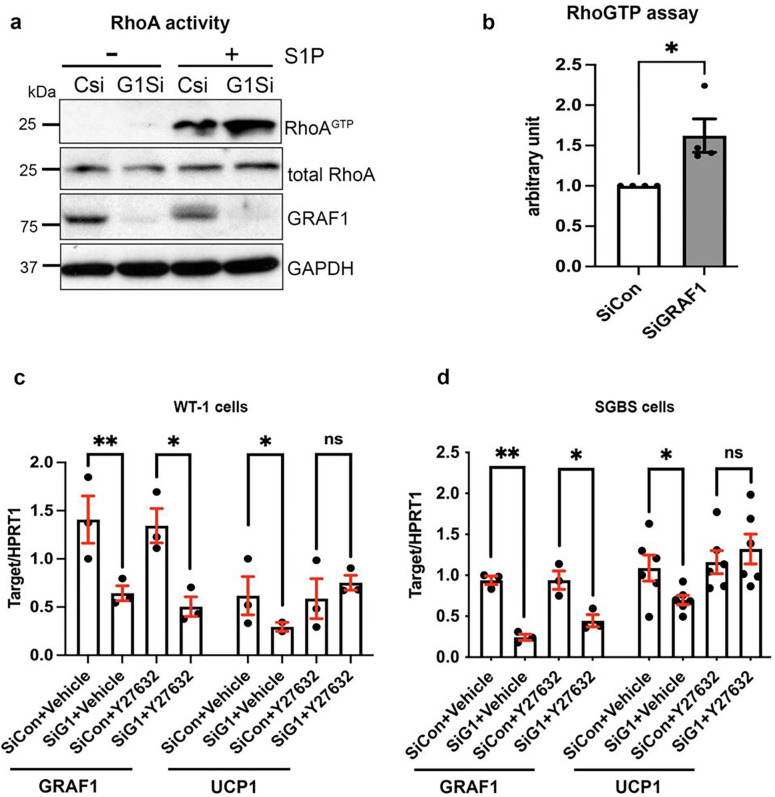
GRAF1 promotes BAT differentiation by limiting RhoA/ROCK signaling. **a and b.** RhoA activity in siRNA transfected WT-1 brown adipocytes was assessed by a standard GST-rhotekin precipitation assay(**a**) and quantified(**b**), n=4 independent experiments. **c and d**. ROCK inhibitor Y-27632 restored UCP-1 expression in GRAF1-depleted WT-1 cells (**c**, n=2–3/group) and in GRAF1-depleted SGBS human brown adipocytes(**d**, n=3–6/group).Data(**b,c,d**) are represented as mean ± SEM, ns, not significant; *P < 0.05; **P < 0.01 by two-tailed student’s t-test.

Because previous studies have shown that the downregulation of RhoA-ROCK signaling is necessary and sufficient to promote adipogenesis, we reasoned that GRAF1 might promote BAT differentiation by controlling this pathway. To test this possibility, we quantified RhoA activity in WT-1 brown adipocytes using a standard GST-rhotekin precipitation assay. As shown in Fig. 5a and b, GRAF1-depleted WT-1 cells exhibited significantly higher levels of RhoA activity in comparison with control siRNA-treated cells following treatment with the RhoA agonist, sphingosine-1-phosphate. Importantly, treatment with the ROCK inhibitor, Y27632 at the onset of differentiation completely restored UCP-1 expression in GRAF1-depleted WT-1 cells (Fig. 5c) and in GRAF1-depleted SGBS human brown adipocytes (Fig. 5d). Collectively, these data indicate that GRAF1 promotes brown adipocyte differentiation by limiting RhoA/ROCK signaling.

## Discussion

Adipose tissue in mammals is a vital component of the energy regulation system and consists of two primary types: white adipocytes and brown adipocytes. White adipocytes are characterized by their large central lipid droplets, serving as reservoirs for storing excess energy in the form of triglycerides. In contrast, brown adipocytes feature small lipid droplets and a high number of mitochondria, enabling them to dissipate energy as heat through a process known as thermogenesis ^39, 40^. The discovery of metabolically active brown adipose tissue (BAT) in healthy adult humans and beige adipocytes within subcutaneous white adipose tissues(scBAT) has generated significant interest in the biology of brown and beige adipocytes due to their unique ability to improve whole-body glucose and lipid metabolism by consuming substantial amounts of blood glucose and lipids. Consequently, identifying molecules and signals that can regulate brown fat differentiation and function has become an attractive target for potential treatments of obesity and diabetes. Our study demonstrates for the first time that GRAF1 regulates brown adipogenesis. Depletion of GRAF1 reduces brown adipocyte differentiation and thermogenesis function in vivo, highlighting its unique role in mediating BAT cell fate.

The differentiation, activation, and maintenance of brown and beige adipocytes are governed by a complex interplay of multiple factors. These factors include endocrine signals such as fibroblast growth factors(FGFs) and bone morphogenic protein factors(BMPs), as well as critical transcription factors like PRDM16, PGC1α, PPARγ, and Foxp1^6, 7, 41–43^. Of particular interest, the RhoA/ROCK pathway has emerged as a key player in regulating adipogenesis. Previous studies have shown that disrupting this pathway, either through the expression of dominant-negative RhoA or the inhibition of ROCK, promotes differentiation in various adipogenic cell types, highlighting the significance of this pathway. Mechanistically, induction of adipocyte differentiation leads to downregulation of RhoA-ROCK signaling, which promotes disassembly of F-actin stress fibers and results in marked changes in cell shape that are thought to be important for lipid droplet accumulation. Depolymerization of F-actin also leads to accumulation of monomeric G-actin, which binds and sequester MRTFs in the cytosol, preventing their nuclear translocation. As MRTFs and their co-factor, SRF repress PPARγ, this critical step allows for the expression of PPARγ and its target genes, facilitating the development and maintenance of adipocyte characteristics during adipogenic differentiation ^15, 16, 44^.

The Rho GTPase can exist in either an inactive GDP-bound or an active GTP-bound form and is modulated by guanine nucleotide exchange factors (GEFs) and GTPase-activating proteins (GAPs) ^45^.The comprehensive understanding of the diverse regulators of Rho GTPase/ROCK in adipose tissues and their in vivo functional consequences remains relatively unexplored. Our previous studies have shown that GRAF1 is a bone fide RhoGAP and plays important role in processes like myoblast fusion, which require GAP-dependent actin remodeling ^28^. Given GRAF1’s established function as a RhoGAP in various tissues, we hypothesized that it might also play a pivotal role in modulating RhoA activity during adipogenesis. Indeed, we observed that GRAF1 is highly and selectively expressed in metabolically active tissues, including brown adipose tissue (BAT), brain, and heart, and its expression profile closely correlated with brown adipocyte differentiation. This association is evidenced by the upregulation of GRAF1 in response to brown adipocyte differentiation medium in various cultured adipocytes and the significant increase in GRAF1 mRNA levels during the maturation stage of BAT in mice, but not in WAT. GRAF1 deficiency, as observed in primary pre-brown adipocyte cultures and in both global and adipose-specific GRAF1-deficient mice, significantly blunted brown and beige adipose differentiation. Furthermore, GRAF1-deficient mice exhibited an inability to efficiently respond to cold challenge-induced thermogenesis. Moreover, development of beige adipocytes in scWAT was compromised in response to browning agent CL316243 stimulation via the β3-adrenergic pathway. Collectively, these data indicate that GRAF1 plays a key cell autonomous role in the development and maturation of beige and brown adipose tissue.

Our mechanistic studies showed that GRAF1 depletion in brown preadipocyte cell line increased Rho activity, suggesting that GRAF1 mediates brown adipocyte differentiation by GAP-dependent Rho GTPase inhibition. Interestingly, GRAF1 also possesses a BAR (Bin/amphiphysin/Rvs) and PH (pleckstrin homology) domain that are involved in sensing and inducing membrane curvature and determining membrane binding specificity. Interestingly, these domains in combination with an isoform-specific hydrophobic segment (found in brain-selective GRAF1a) were previously reported to drive GRAF1a association with lipid droplets, an event that promoted lipid droplet clustering and reduced lipolysis ^46, 47^. While our current study focused on mouse GRAF1.2 (the ortholog of GRAF1b in humans) which does not contain this hydrophobic region, future studies are warranted to determine the extent to which GRAF1.2/GRAF1b may regulate adipocyte lipid droplet homeostasis.

Previous studies have demonstrated that p-190 B is a RhoGAP can that shift myogenesis towards adipogenesis by inhibiting Rho GTPase in adipogenic precursor cells^17–19^. Another RhoGAP, DLC1, has been reported to promote both white and brown adipocyte differentiation and to provide a molecular link between PPARγ and Rho signaling pathways ^20^. However, it’s important to note that while p-190 B and DLC1 play significant roles in regulating adipocyte differentiation, neither of these RhoGAPs exhibits selective induction of brown adipocyte differentiation. In our current study, we present the unique contribution of GRAF1, a RhoGAP, to the differentiation of brown and beige adipocytes. This distinctive function differentiates GRAF1 from other RhoGAPs. Combining in vitro investigations across various cell types with in vivo experiments in GRAF1 knockout mouse models, we have demonstrated that GRAF1 promotes classical brown fat maturation and the development of beige adipocytes in scWAT. This regulation, at least partly, occurs through GRAF1’s ability to suppress RhoA activity, thereby limiting the RhoA-ROCK signal pathway and promoting brown adipocyte differentiation and maturation. Future research may elucidate the potential for manipulating GRAF1 expression and its GAP activity to improve metabolic profiles, holding promise for the treatment of metabolic diseases, including obesity and insulin resistance.

## Methods

### Animals

GRAF1 gene trap mice were generated and obtained from the Texas A&M Institute for Genomic Medicine (College Station, TX) and were described previously ^28^. ES cells carrying Arhgap26 targeted knockout first conditional-ready alleles were obtained from EuMMCR. Germline transmission of the allele was confirmed from F0 chimeric mice with C57BL/6 genetic background. Next, we crossed Flp recombinase transgenic mice with F1 to generate GRAF1 floxed mice. Finally, we established adipose specific GRAF1 knockout mouse line (GRAF1^AKO^) by crossing Adiponectin Cre line (010803, Jackson Lab ^36^) with GRAF1 floxed mice. All mice were housed in pathogen-free facilities under a 12-hour light/dark cycle with unrestricted access to food and water. Animals were treated in accordance with the approved protocol of the University of North Carolina (Chapel Hill, NC) Institutional Animal Care and Use Committee, which is in compliance with the standards outlined in the guide for the Care and Use of Laboratory Animals. All methods are reported in accordance with ARRIVE (Animal Research: Reporting of In Vivo Experiments) guidelines.

To induce GRAF1 knockout *in vivo*, 80mg of Tamoxifen power (Sigma, T5648) was dissolved in 750ul 100% ethanol(molecular biology grade) (ThermoFisher Scientific, T038181000) for 40 mins at 40°C, then was mixed with 3.25 ml filtered corn oil(Sigma, C8267) to make final concentration of 20mg/ml. Tamoxifen was administered at 100mg/kg via oral gavage once every 24 hours for a total of 5 consecutive days. 1 week later, mice were subject to experiment treatment. To induce the browning program of white fat depots, control and Adcre-GRAF1ko (GRAF1^AKO^) mice received daily intraperitoneal injections of β3-adrenergic agonist CL316243 at 1mg/kg (Sigma, C5976)for 10 days as described previously ^48^.

### Cold exposure

To assess sensitivity to cold exposure, we monitored rectal temperature at hourly intervals following the placement of mice in a cold room maintained at 6–8 °C. The mice underwent an overnight fast, which continued throughout the duration of the cold exposure. Subsequent to the cold exposure period, mice were humanely euthanized by CO2 inhalation.

### Cell culture and differentiation

Human Simpson-Golabi-Behmel syndrome (SGBS) preadipocyte line was provided by Dr. Martin Wabitsch (Ulm University Medical Center, Germany). The cells were maintained in DMEM/F12 (ThermoFisher Scientific, 11330–032) supplemented with 10% fetal calf serum(FCS), biotin 3.3 mM, pantothenate 1.7 mM and antibiotics. When cells were 90–100% confluence, SGBS cells were induced in brown adipocyte differentiation medium for 4 days (DMEM/F12, 3.3 mM biotin, 1.7 mM pantothenate, transferrin 10 μg/ml, 430 nMol insulin, 1nMol T3, 1μMol dexamethasone, 0.5mMol IBMX, 2μMol Rosiglitazone and antibiotics). Then cell medium was changed to brown adipocyte maintenance medium every 4 days for 8 days (DMEM/F12, 3.3 mM biotin, 1.7 mM pantothenate, transferrin 10 μg/ml, 430 nMol insulin, 1nMol T3 and antibiotics).

### Isolation and differentiation of Stromal Vascular Fraction (SVF) Cells

Subcutaneous WAT (inguinal WAT) or interscapular BAT tissues were used for Beige cell source, and followed a previously described isolation and differentiation method^49^. In brief, tissues were digested and centrifuged to get SVF cells. SVF cells were cultured to 100% confluence in complete medium (DMEM/F12 containing 10% FPS and P/S). SVF cells were cultured for 2 days in induction medium (complete medium plus 5μg/ml insulin, 1 nM T3, 125 μM Indomethacin, 2 μg/ml Dexamethasone, 0.5 mM IBMX, 0.5 μM Rosiglitazone). Then cell medium was changed to maintenance medium (complete medium plus 5μg/ml insulin, 1 nM T3) with 0.5 μM rosiglitazone for 2 days. At Day 4, cell medium was changed to maintenance medium with 1 μM rosiglitazone for 2 days.

WT-1 cells were provided by Dr. Yu-Hua Tseng (Joslin Diabetes Center, Harvard Medical School Affiliate, Boston, MA). Cells were cultured and induced differentiation with the same medium as SVF cells.

### SiRNA treatment

Cells were plated at 1.2 × 105/ 2 ml/well in 6 well plate. Next day, cells were transfected GRAF1 targeting siRNA or GFP targeting siRNA (final concentration 10 nM) by Lipofectamine^®^ RNAiMAX(ThermoFisher Scientific, 13778075) following the manufacturer’s instructions for 72 hours. GRAF1 Stealth RNAi^™^ siRNA was synthesized by ThermoFisher with the following sequences: 5’-CGGAAGUUUGCAGAUUCCUUAAAUG-3’ and 5’-CAUUUAAGGAAUCUGCAAACUUCCG-3’. GFP stealth siRNA was used as Control with the following sequence: 5’-GGUGCGCUCCUGGACGUAGCC[dT][dT]-3’ 5’-GGCUACGUCCAGGAGCGCACC[dT][dT]-3’.

### Real time PCR analysis

Total RNA was isolated from homogenized whole mouse tissues or cell cultures using RNeasy Mini Kit (Qiagen, 74106) according to manufacturer’s instructions. After homogenizing, samples were placed on ice to remove lipid layer. Complimentary DNA (cDNA) was obtained from 1 μg of RNA isolated using the iScript cDNA Synthesis Kit (Bio-Rad, 1708897), and cDNA was used for qPCR with iTaq Universal SYBR Green Supermix kit (Bio-Rad, 1725124). The relative gene expression levels were calculated using delta-delta Ct method, also known as the 2– ΔΔCt method. Primer sequences were in Supplemental Table 1.

### RhoA activity assay

WT-1 cells were serum starved overnight prior to treatment with S1P for indicated times. Rho activity was measured by GST-Rhotekin pulldown assay as previously described ^50^. Cell lysates were rotated with 40 μg of a GST-rhotekin Rho binding domain fusion protein immobilized to glutathione-Sepharose 4B beads (Cytiva, 17075601) for 15 min at 4°C in binding buffer (50 mM Tris, pH 7.6, 500 mM NaCl, 0.1% SDS, 0.5% deoxycholate, 1% Triton X-100, 0.5 mM MgCl2). Beads were precipitated and washed three times (50 mM Tris, pH 7.6, 150 mM NaCl, 1% Triton X-100, 0.5 mM MgCl2) and resuspended in 2 × Laemmli buffer. Proteins were separated on a 15% SDS–PAGE and transferred to 0.2 μm PVDF membrane (Bio-Rad, 1620177). After blocking in 5% bovine serum albumin/TBST (20 mM Tris–HCl, 500 mM NaCl, 0.05% Tween-20, pH 7.4) for 1 hour at room temperature, blots were probed with 2 μg/ml anti-RhoA (26C4) (Santa Cruz Biotechnology, SC418) overnight at 4°C. Loading controls (typically 10%) were taken from each lysate sample prior to pull downs.

### Western blotting

To examine protein levels, lysates from cells or tissues were prepared by lysing in a modified RIPA buffer with 1x HALT phosphatase & protease inhibitor cocktail(ThermoFisher Scientific, 78438 and 78427). Protein concentration was determined by using a colorimetric BCA assay (Pierce, 23227). Lysates were electrophoresed on SDS–polyacrylamide gel, transferred to nitrocellulose and immunoblotted with specific antibodies overnight at 4°C as indicated using a 1:1000 dilution. The following primary antibodies were used in western blot: GAPDH(cell signaling technology, 5174S), b-actin(cell signaling technology, 3700S), α-Tubulin(Sigma, T6199), RhoA(Santa Cruz, SC-418). Rabbit anti-GRAF1 polyclonal antibody is homemade antibody in our lab. Blots were washed in TBST (TBS plus 0.1%Tween20) followed by incubation with horseradish peroxidase conjugated antibody at a 1/1,000 dilution. Blots were visualized after incubation with chemiluminescence reagents (ThermoFisher Scientific, 32106).

### Oil red O staining and quantification

Cells were rinsed with PBS and then fixed in 4% PFA for 30 minutes. Then cells were left in the air until completely dry. Oil Red-O working solution (0.3%) was freshly prepared and stained cells on the shaker for 10 minutes. Then cells were rinsed with PBS 3 times and used for imaging. After imaging, liquid was removed from cells completely. To elute the oil red O dye, 100% isopropanol was added to the plates. The plates were incubated for 10 min at room temperature on an orbital shaker. Absorption was measured at 518 nm on a plate reader.

### Statistics

Unless stated otherwise, all data represented at least three individual experiments and presented as means ± standard error of the mean (SEM). Means of normally distributed data were compared by two-tailed Student’s t-test, one-way ANOVA (followed by Tukey’s post-hoc correction) or linear regression where indicated and statistical significance was reported as p-values. A p-value < 0.05 was considered significant. Sample sizes were chosen based on an extensive literature search and standard exclusion criterion of two standard deviations from the mean were applied.

## Data Availability

All data generated or analysed during this study are included in this published article and its supplementary information files.

## References

[1] Sanchez-Gurmaches J and Guertin DA. Adipocytes arise from multiple lineages that are heterogeneously and dynamically distributed. Nat Commun. 2014;5:4099. 24942009 10.1038/ncomms5099 PMC4066194

[2] Huang P, Schulz TJ, Beauvais A, Tseng YH and Gussoni E. Intramuscular adipogenesis is inhibited by myo-endothelial progenitors with functioning Bmpr1a signalling. Nat Commun. 2014;5:4063. 24898859 10.1038/ncomms5063 PMC4084855

[3] Ahmadian M, Suh JM, Hah N, Liddle C, Atkins AR, Downes M and Evans RM. PPARγ signaling and metabolism: the good, the bad and the future. Nat Med. 2013;19:557–66. 23652116 10.1038/nm.3159 PMC3870016

[4] Hu E, Liang P and Spiegelman BM. AdipoQ is a novel adipose-specific gene dysregulated in obesity. J Biol Chem. 1996;271:10697–703. 8631877 10.1074/jbc.271.18.10697

[5] Zhou Z, Yon Toh S, Chen Z, Guo K, Ng CP, Ponniah S, Lin SC, Hong W and Li P. Cidea-deficient mice have lean phenotype and are resistant to obesity. Nature genetics. 2003;35:49–56. 10.1038/ng1225 12910269

[6] Betz MJ and Enerback S. Targeting thermogenesis in brown fat and muscle to treat obesity and metabolic disease. Nat Rev Endocrinol. 2018;14:77–87. 29052591 10.1038/nrendo.2017.132

[7] Schulz TJ and Tseng YH. Brown adipose tissue: development, metabolism and beyond. Biochem J. 2013;453:167–78. 23805974 10.1042/BJ20130457 PMC3887508

[8] Elsen M, Raschke S, Tennagels N, Schwahn U, Jelenik T, Roden M, Romacho T and Eckel J. BMP4 and BMP7 induce the white-to-brown transition of primary human adipose stem cells. American journal of physiology Cell physiology. 2014;306:C431–40. 24284793 10.1152/ajpcell.00290.2013

[9] Gustafson B, Hammarstedt A, Hedjazifar S, Hoffmann JM, Svensson PA, Grimsby J, Rondinone C and Smith U. BMP4 and BMP Antagonists Regulate Human White and Beige Adipogenesis. Diabetes. 2015;64:1670–81. 25605802 10.2337/db14-1127

[10] Tseng YH, Kokkotou E, Schulz TJ, Huang TL, Winnay JN, Taniguchi CM, Tran TT, Suzuki R, Espinoza DO, Yamamoto Y, Ahrens MJ, Dudley AT, Norris AW, Kulkarni RN and Kahn CR. New role of bone morphogenetic protein 7 in brown adipogenesis and energy expenditure. Nature. 2008;454:1000–4. 18719589 10.1038/nature07221 PMC2745972

[11] Blázquez-Medela AM, Jumabay M and Boström KI. Beyond the bone: Bone morphogenetic protein signaling in adipose tissue. Obesity reviews: an official journal of the International Association for the Study of Obesity. 2019;20:648–658. 30609449 10.1111/obr.12822 PMC6447448

[12] Sharma A, Huard C, Vernochet C, Ziemek D, Knowlton KM, Tyminski E, Paradis T, Zhang Y, Jones JE, von Schack D, Brown CT, Milos PM, Coyle AJ, Tremblay F and Martinez RV. Brown fat determination and development from muscle precursor cells by novel action of bone morphogenetic protein 6. PLoS One. 2014;9:e92608. 24658703 10.1371/journal.pone.0092608 PMC3962431

[13] Wei L, Surma M, Yang Y, Tersey S and Shi J. ROCK2 inhibition enhances the thermogenic program in white and brown fat tissue in mice. Faseb j. 2020;34:474–493. 31914704 10.1096/fj.201901174RR PMC6956740

[14] Khan AU, Qu R, Fan T, Ouyang J and Dai J. A glance on the role of actin in osteogenic and adipogenic differentiation of mesenchymal stem cells. Stem cell research & therapy. 2020;11:283. 32678016 10.1186/s13287-020-01789-2 PMC7364498

[15] McDonald ME, Li C, Bian H, Smith BD, Layne MD and Farmer SR. Myocardin-related transcription factor A regulates conversion of progenitors to beige adipocytes. Cell. 2015;160:105–18. 25579684 10.1016/j.cell.2014.12.005 PMC4384505

[16] Rosenwald M, Efthymiou V, Opitz L and Wolfrum C. SRF and MKL1 Independently Inhibit Brown Adipogenesis. PLoS One. 2017;12:e0170643. 28125644 10.1371/journal.pone.0170643 PMC5268445

[17] Inagaki T, Sakai J and Kajimura S. Transcriptional and epigenetic control of brown and beige adipose cell fate and function. Nat Rev Mol Cell Biol. 2016;17:480–95. 27251423 10.1038/nrm.2016.62 PMC4956538

[18] Seale P, Bjork B, Yang W, Kajimura S, Chin S, Kuang S, Scime A, Devarakonda S, Conroe HM, Erdjument-Bromage H, Tempst P, Rudnicki MA, Beier DR and Spiegelman BM. PRDM16 controls a brown fat/skeletal muscle switch. Nature. 2008;454:961–7. 18719582 10.1038/nature07182 PMC2583329

[19] Yin H, Pasut A, Soleimani VD, Bentzinger CF, Antoun G, Thorn S, Seale P, Fernando P, van Ijcken W, Grosveld F, Dekemp RA, Boushel R, Harper ME and Rudnicki MA. MicroRNA-133 controls brown adipose determination in skeletal muscle satellite cells by targeting Prdm16. Cell Metab. 2013;17:210–24. 23395168 10.1016/j.cmet.2013.01.004 PMC3641657

[20] Sim CK, Kim SY, Brunmeir R, Zhang Q, Li H, Dharmasegaran D, Leong C, Lim YY, Han W and Xu F. Regulation of white and brown adipocyte differentiation by RhoGAP DLC1. PLoS One. 2017;12:e0174761. 28358928 10.1371/journal.pone.0174761 PMC5373604

[21] Taylor JM, Macklem MM and Parsons JT. Cytoskeletal changes induced by GRAF, the GTPase regulator associated with focal adhesion kinase, are mediated by Rho. J Cell Sci. 1999;112 (Pt 2):231–42. 9858476 10.1242/jcs.112.2.231

[22] Taylor JM, Hildebrand JD, Mack CP, Cox ME and Parsons JT. Characterization of graf, the GTPase-activating protein for rho associated with focal adhesion kinase. Phosphorylation and possible regulation by mitogen-activated protein kinase. J Biol Chem. 1998;273:8063–70. 9525907 10.1074/jbc.273.14.8063

[23] Doherty JT, Lenhart KC, Cameron MV, Mack CP, Conlon FL and Taylor JM. Skeletal muscle differentiation and fusion are regulated by the BAR-containing Rho-GTPase-activating protein (Rho-GAP), GRAF1. J Biol Chem. 2011;286:25903–21. 21622574 10.1074/jbc.M111.243030 PMC3138304

[24] Sanchez-Gurmaches J, Hung CM, Sparks CA, Tang Y, Li H and Guertin DA. PTEN loss in the Myf5 lineage redistributes body fat and reveals subsets of white adipocytes that arise from Myf5 precursors. Cell Metab. 2012;16:348–62. 22940198 10.1016/j.cmet.2012.08.003 PMC3488151

[25] Gensch N, Borchardt T, Schneider A, Riethmacher D and Braun T. Different autonomous myogenic cell populations revealed by ablation of Myf5-expressing cells during mouse embryogenesis. Development. 2008;135:1597–604. 18367555 10.1242/dev.019331

[26] Sordella R, Jiang W, Chen GC, Curto M and Settleman J. Modulation of Rho GTPase signaling regulates a switch between adipogenesis and myogenesis. Cell. 2003;113:147–58. 12705864 10.1016/s0092-8674(03)00271-x

[27] Murholm M, Dixen K, Qvortrup K, Hansen LH, Amri EZ, Madsen L, Barbatelli G, Quistorff B and Hansen JB. Dynamic regulation of genes involved in mitochondrial DNA replication and transcription during mouse brown fat cell differentiation and recruitment. PLoS One. 2009;4:e8458. 20107496 10.1371/journal.pone.0008458 PMC2809086

[28] Lenhart KC, Becherer AL, Li J, Xiao X, McNally EM, Mack CP and Taylor JM. GRAF1 promotes Ferlin-dependent myoblast fusion. Dev Biol. 2014;393:298–311. 25019370 10.1016/j.ydbio.2014.06.025 PMC4535172

[29] Giralt M, Martin I, Iglesias R, Viñas O, Villarroya F and Mampel T. Ontogeny and perinatal modulation of gene expression in rat brown adipose tissue. Unaltered iodothyronine 5’-deiodinase activity is necessary for the response to environmental temperature at birth. European journal of biochemistry. 1990;193:297–302. 2171932 10.1111/j.1432-1033.1990.tb19336.x

[30] Wang QA, Tao C, Gupta RK and Scherer PE. Tracking adipogenesis during white adipose tissue development, expansion and regeneration. Nat Med. 2013;19:1338–44. 23995282 10.1038/nm.3324 PMC4075943

[31] Kodde A, Engels E, Oosting A, van Limpt K, van der Beek EM and Keijer J. Maturation of White Adipose Tissue Function in C57BL/6j Mice From Weaning to Young Adulthood. Front Physiol. 2019;10:836. 31354508 10.3389/fphys.2019.00836 PMC6629938

[32] Liu X, Rossmeisl M, McClaine J, Riachi M, Harper ME and Kozak LP. Paradoxical resistance to diet-induced obesity in UCP1-deficient mice. J Clin Invest. 2003;111:399–407. 12569166 10.1172/JCI15737 PMC151850

[33] Ellis JM, Li LO, Wu PC, Koves TR, Ilkayeva O, Stevens RD, Watkins SM, Muoio DM and Coleman RA. Adipose acyl-CoA synthetase-1 directs fatty acids toward beta-oxidation and is required for cold thermogenesis. Cell Metab. 2010;12:53–64. 20620995 10.1016/j.cmet.2010.05.012 PMC2910420

[34] Hui S, Ghergurovich JM, Morscher RJ, Jang C, Teng X, Lu W, Esparza LA, Reya T, Le Z, Yanxiang Guo J, White E and Rabinowitz JD. Glucose feeds the TCA cycle via circulating lactate. Nature. 2017;551:115–118. 29045397 10.1038/nature24057 PMC5898814

[35] Dufau J, Shen JX, Couchet M, De Castro Barbosa T, Mejhert N, Massier L, Griseti E, Mouisel E, Amri EZ, Lauschke VM, Rydén M and Langin D. In vitro and ex vivo models of adipocytes. American journal of physiology Cell physiology. 2021;320:C822–c841. 33439778 10.1152/ajpcell.00519.2020

[36] Eguchi J, Wang X, Yu S, Kershaw EE, Chiu PC, Dushay J, Estall JL, Klein U, Maratos-Flier E and Rosen ED. Transcriptional control of adipose lipid handling by IRF4. Cell Metab. 2011;13:249–59. 21356515 10.1016/j.cmet.2011.02.005 PMC3063358

[37] Schlaepfer IR and Joshi M. CPT1A-mediated Fat Oxidation, Mechanisms, and Therapeutic Potential. Endocrinology. 2020;161. 10.1210/endocr/bqz046 31900483

[38] Giudice J and Taylor JM. Muscle as a paracrine and endocrine organ. Curr Opin Pharmacol. 2017;34:49–55. 28605657 10.1016/j.coph.2017.05.005 PMC5808999

[39] Richard AJ, White U, Elks CM and Stephens JM. Adipose Tissue: Physiology to Metabolic Dysfunction. In: Feingold K. R., Anawalt B., Blackman M. R., Boyce A., Chrousos G., Corpas E., de Herder W. W., Dhatariya K., Dungan K., Hofland J., Kalra S., Kaltsas G., Kapoor N., Koch C., Kopp P., Korbonits M., Kovacs C. S., Kuohung W., Laferrère B., Levy M., McGee E. A., McLachlan R., New M., Purnell J., Sahay R., Shah A. S., Singer F., Sperling M. A., Stratakis C. A., Trence D. L. and Wilson D. P., eds. Endotext South Dartmouth (MA): MDText.com, Inc. Copyright © 2000–2023, MDText.com, Inc.; 2000.

[40] Sakers A, De Siqueira MK, Seale P and Villanueva CJ. Adipose-tissue plasticity in health and disease. Cell. 2022;185:419–446. 35120662 10.1016/j.cell.2021.12.016 PMC11152570

[41] Harms MJ, Ishibashi J, Wang W, Lim HW, Goyama S, Sato T, Kurokawa M, Won KJ and Seale P. Prdm16 is required for the maintenance of brown adipocyte identity and function in adult mice. Cell Metab. 2014;19:593–604. 24703692 10.1016/j.cmet.2014.03.007 PMC4012340

[42] Liu P, Huang S, Ling S, Xu S, Wang F, Zhang W, Zhou R, He L, Xia X, Yao Z, Fan Y, Wang N, Hu C, Zhao X, Tucker HO, Wang J and Guo X. Foxp1 controls brown/beige adipocyte differentiation and thermogenesis through regulating β3-AR desensitization. Nat Commun. 2019;10:5070. 31699980 10.1038/s41467-019-12988-8 PMC6838312

[43] Liang H and Ward WF. PGC-1alpha: a key regulator of energy metabolism. Advances in physiology education. 2006;30:145–51. 17108241 10.1152/advan.00052.2006

[44] Cristancho AG and Lazar MA. Forming functional fat: a growing understanding of adipocyte differentiation. Nat Rev Mol Cell Biol. 2011;12:722–34. 21952300 10.1038/nrm3198 PMC7171550

[45] Etienne-Manneville S and Hall A. Rho GTPases in cell biology. Nature. 2002;420:629–35. 12478284 10.1038/nature01148

[46] Lucken-Ardjomande Häsler S, Vallis Y, Jolin HE, McKenzie AN and McMahon HT. GRAF1a is a brain-specific protein that promotes lipid droplet clustering and growth, and is enriched at lipid droplet junctions. J Cell Sci. 2014;127:4602–19. 25189622 10.1242/jcs.147694 PMC4215711

[47] Lundmark R, Doherty GJ, Howes MT, Cortese K, Vallis Y, Parton RG and McMahon HT. The GTPase-activating protein GRAF1 regulates the CLIC/GEEC endocytic pathway. Current biology: CB. 2008;18:1802–8. 19036340 10.1016/j.cub.2008.10.044 PMC2726289

[48] Dempersmier J, Sambeat A, Gulyaeva O, Paul SM, Hudak CS, Raposo HF, Kwan HY, Kang C, Wong RH and Sul HS. Cold-inducible Zfp516 activates UCP1 transcription to promote browning of white fat and development of brown fat. Molecular cell. 2015;57:235–46. 25578880 10.1016/j.molcel.2014.12.005 PMC4304950

[49] Aune UL, Ruiz L and Kajimura S. Isolation and differentiation of stromal vascular cells to beige/brite cells. Journal of visualized experiments: JoVE. 2013. 10.3791/50191 PMC3641667 23568137

[50] Bai X, Lenhart KC, Bird KE, Suen AA, Rojas M, Kakoki M, Li F, Smithies O, Mack CP and Taylor JM. The smooth muscle-selective RhoGAP GRAF3 is a critical regulator of vascular tone and hypertension. Nat Commun. 2013;4:2910. 24335996 10.1038/ncomms3910 PMC4237314

